# Effects of running-based versus body-weight-based high-intensity interval training on physical fitness in healthy adolescents

**DOI:** 10.3389/fphys.2023.1060216

**Published:** 2023-03-10

**Authors:** Zhen Li, Yang Liu, Xiaowei Han, Zhixiong Zhou

**Affiliations:** ^1^ Provincial University Key Laboratory of Sport and Health Science, School of Physical Education and Sport Science, Fujian Normal University, Fuzhou, China; ^2^ Hebei Institute of International Business and Economics, Qinhuangdao, China; ^3^ Faculty of Education, Beijing Normal University, Beijing, China; ^4^ School of Physical Education and Coaching Science, Capital University of Physical Education and Sports, Beijing, China

**Keywords:** HIIT, middle school students, VO2max, muscle fitness, school environment

## Abstract

**Objectives:** High-intensity interval training improves aerobic endurance, but the effectiveness of different training protocols is unclear. This study compared the effects of running-based high-intensity interval training (R-HIIT) and body weight-based high-intensity interval training (B-HIIT) on physical fitness in adolescents.

**Methods:** This was a pre-and post-test quasi-experimental design in which a seventh-grade natural class was randomly selected from three homogeneous middle schools, and then the three natural classes were randomly divided into three groups: the R-HIIT group (*n* = 54), the B-HIIT group (*n* = 55), and the control group (Con, *n* = 57). Both intervention groups exercised twice a week for 12 weeks with a 2:1 (1 min:30 s) load-interval ratio and exercise intensity controlled at 70%–85% maximum heart rate. R-HIIT was in the form of running, and B-HIIT was in the form of resistance exercises using the participants’ body weight. The control group was instructed to continue their normal behavior. cardiorespiratory fitness, muscle strength and endurance, and speed were measured before and after the intervention. Statistical differences between and within groups were determined using repeated measures analysis of variance.

**Results:** Compared to the baseline, both the R-HIIT and B-HIIT intervention groups significantly improved CRF, muscle strength, and speed (*p* < 0.05). The B-HIIT group was significantly better than R-HIIT in improving CRF (4.48 mL/kg/min vs 3.34 mL/kg/min, *p* < 0.05), and only the B-HIIT improved sit-up muscle endurance (η_p_^2^ = 0.30, *p* < 0.05).

**Conclusion:** The B-HIIT protocol was significantly more effective than the R-HIIT protocol in developing CRF and improving muscle health indicators.

## 1 Introduction

Participation in regular physical activities (PA) promotes the physical (Lubans et al., 2022), psychological (Lubans et al., 2016), and cognitive (Donnelly et al., 2016) health of children and adolescents. The World Health Organization recommends that children and adolescents should participate in 60 min of moderate to vigorous physical activity every day to obtain health benefits (Chaput et al., 2020). However, data show that less than 20% of children and adolescents worldwide, and only 6% in Asia, meet the recommended levels (Guthold et al., 2020; Loo et al., 2022). Furthermore, the global spread of COVID-19 has exacerbated the trend of declining activity levels among children and adolescents (Farooq et al., 2018; Sa et al., 2020). There is a strong association between a lack of physical activity and high levels of obesity and elevated blood pressure in children, which, if not treated promptly, significantly increases the risk of cardiovascular disease, cancer, and type 2 diabetes in adulthood (Paulino da Silva Bento et al., 2021).

School physical education (PE) lessons provide opportunities for children and adolescents to participate in physical activity and promote their health, but, due to curriculum and personal reasons, each student only participates in exercise between 18% and 50% of the total time in a PE lesson (Gehris et al., 2012; Hollis et al., 2017; Breithecker et al., 2021; Bossmann et al., 2022). Therefore, traditional interventions to improve physical health, such as long-term moderate-intensity continuous training (MICT), do not seem suitable for the school environment (Paulino da Silva Bento et al., 2021). In addition to the time factor, the intensity of PE lessons is insufficient, weakening their potential health benefits (Hollis et al., 2017; Breithecker et al., 2021; Bossmann et al., 2022). Therefore, it is necessary to explore and develop alternate ways to attract children and adolescents to realize the many health benefits related to regular physical activity.

Researchers and the public have paid much attention to high-intensity interval training (HIIT) in recent years because it can produce physiological benefits similar to MICT in a shorter time (Thompson, 2022). HIIT programs typically include relatively short but vigorous exercise—e.g., exercise achieving >85% of maximum heart rate (HRmax)—with active rest and recovery periods between intervals (Lubans et al., 2022). The effectiveness of a range of HIIT programs in improving various health indicators, including body composition, cardiorespiratory fitness, and cardiometabolic fitness, has been well established in children and adolescents (Logan et al., 2014; Costigan et al., 2015; Eddolls et al., 2017; Yin et al., 2020). However, considering that the strenuousness of HIIT may cause unhappiness (Ekkekakis et al., 2011), some experts believe that traditional interventions aimed at an increasing activity that rely on motivation and social support are largely ineffective, with the result that most people will not or cannot actively participate (Biddle and Batterham, 2015). In view of these factors, relying on the motivation and self-discipline of young people to participate in HIIT may not be sufficient to carry out health intervention on a large scale.

According to Beets et al. (2016), embedding HIIT into an organized, weekly period, located in an environment with the greatest contact with other children (such as school PE class), may overcome the past limitations of HIIT to carry out large-scale intervention and can also make up for the shortcomings of the short participation time and low intensity of traditional PE lessons. In this context, several studies have incorporated HIIT into school PE lessons (Bauer et al., 2022; Cao et al., 2022b) or other school activities (Engel et al., 2019), and the results have shown improved cardiac metabolic health and neuromuscular adaptation (Bauer et al., 2022). Other studies showed improved health-related parameters through shorter intervention time (Cao et al., 2022b; Popowczak et al., 2022), and HIIT exercises were found to be more enjoyable than traditional methods such as MICT (Bond et al., 2015; Malik et al., 2017).

Despite these prospects, many of these studies pay attention to the effect of HIIT on cardiorespiratory fitness (CRF) and cardiovascular metabolism but do not assess the impact on musculoskeletal health. The current international consensus is that it is important to include musculoskeletal health in the adolescent health assessment (Janz et al., 2021). Second, although the effectiveness of HIIT in a school environment has been proven, most studies have only used the traditional strategy of running-based high-intensity interval training (R-HIIT) (Costigan et al., 2015; Bond et al., 2017; Meng et al., 2022), which is a simple and easy assessment, but may not promote the development of other health indicators such as muscle strength (Hulteen et al., 2018). In addition, R-HIIT may require specialized equipment like treadmills and power bicycles, and/or facilities such as gyms and playgrounds, which may be impractical for some schools (Kennedy et al., 2020).

Body weight-based high-intensity interval training (B-HIIT) has received a lot of attention in recent years because it does not require specialized equipment and can be performed with minimal space. Evidence has shown that, under proper supervision, resistance exercises based on one’s own weight, do not negatively impact the growth and development of children and adolescents and can also improve related health indicators and reduce the risk of injury (Behm et al., 2008; Faigenbaum et al., 2009). Some studies have shown that B-HIIT is better than R-HIIT at improving health-related indicators in children and adolescents (Lubans et al., 2022), but others have come to the opposite conclusion (Engel et al., 2019). Therefore, it is unclear whether B-HIIT can be successfully used as a health promotion program with children and adolescents, especially in school settings.

Furthermore, many current health promotion initiatives for children and adolescents emphasize increasing PA levels, and knowledge of the factors influencing PA levels is limited to psychological, social, and environmental aspects (Dobbins et al., 2013; Gorga et al., 2016; Owen et al., 2017; Messing et al., 2019), omitting the potential influence of intrinsic biological control on normal physical activity (Beck et al., 2022). In order to maintain a level of PA or energy expenditure that is generally steady over time, the ActivityStat hypothesis hypothesizes that an imposed increase or decrease in PA in one domain can induce a compensatory adjustment in the opposite direction in another domain (Gomersall et al., 2013). This phenomenon has been confirmed in several intervention studies (Aburto et al., 2011; Haapala et al., 2017): school-based interventions have shown a slight or moderate effect of increasing PA within the school setting, but little change in overall levels of PA due to the use of compensatory mechanisms outside the school setting. However, some studies have also found that children and adolescents who regularly participate in organized sports do not show indicators of compensatory behavior (Spengler et al., 2015). It is therefore suspected that whether PA interventions induce “compensatory behaviors” in adolescents depends on the mode and duration of the intervention (Beck et al., 2022). Little is known about changes in PA in HIIT-influenced adolescents over 1-day or multiple-day intervals (Lee et al., 2021).

Given that adolescent health is directly tied to both increasing daily PA levels and improving adolescent physical fitness levels, this study had two objectives. The primary objective of this was to investigate the effects of two HIIT protocols (R-HIIT and B-HIIT) on adolescent physical fitness in order to establish an intervention framework that would more effectively promote healthy adolescent development. We hypothesize 1 that B-HIIT is better than R-HIIT in improving the physical fitness index of adolescents because it emphasizes using and controlling one’s weight, which can activate more muscles than R-HIIT and impose significant physiological stimulation on the body (Sumpena and Sidik, 2016; Ricci et al., 2020). The secondary objective is to evaluate the changes in daily PA level of adolescents during the intervention period. According to the ActivityStat hypothesis, we hypothesized 2 that the implementation of HIIT intervention in PE class will lead to a decrease in daily PA level of adolescents.

## 2 Methods

### 2.1 Study design and participants

This was a pre-and post-test study using a quasi-experimental design with the addition of a control group. The 12-week intervention was conducted from September to December 2018. Based on our school-based recruitment through consultations with nearby pediatricians, convenience sampling was used. The following factors guided our decision to choose three middle schools in Beijing, China, as intervention centers: The schools were all coeducational, had comparable teacher-student ratios, teacher qualification standards, and teacher experience levels, were all public schools that followed the same educational curriculum, and each student in each school had similar household income and parental education levels. In addition, all the schools had adequate space for both indoor and outdoor physical activity.

The inclusion criteria for subjects in this study were 1) age 11–13, as this age group was underrepresented in previous studies, 2) no regular physical activity other than physical education classes within 3 months before the experiment, and 3) no contraindication to any exercise. Criteria for exclusion were: 1) exercise contraindications or restrictions; 2) acute illness or sports injuries; 3) psychiatric or other psychological disorders, and 4) congenital or acquired heart disease. All subjects were informed of the risks and requirements of the training program, and we obtained signed informed consent from the students and their guardians before the start of the study. The trial was approved by the Ethics Committee of the Capital Institute of Sports (code: 201712001, approval date: 2017/12/26) and registered in the Chinese Clinical Trial Registry (registration number: ChiCTR-IOR-17010435).

In this research, a simple randomization method was used and SPSS software was utilized to create random numbers using random sampling. In each of the three intervention centers, one natural class of seventh graders was chosen at random, and the three natural classes were then divided into three groups at random: R-HIIT group (*n* = 54), B-HIIT group (*n* = 55), and control group (Con, *n* = 57). To mitigate potential confounding effects between the intervention and control groups, randomization was not conducted at the individual level within the schools (Weston et al., 2021). The random assignment form was duplicated three times, with one copy each going to the statistician, the project manager, and the principal. After allocation, participants and data analysts were made blind. Participants in the intervention received specialized training for each of the exercise intervention groups. The independent researcher was in charge of statistical data analysis and made sure that he or she was not aware of how the interventions were distributed in particular.

### 2.2 Study measurements

#### 2.2.1 Anthropometry

We measured the height and weight of each student before and after the intervention using a body composition analyzer (InBody J20, BIOSPACE, Seoul, Korea), which has been shown to have strong validity in Asian children (Lim et al., 2009) and has been used in large intervention trials with children (Kriemler et al., 2010). Body mass index (BMI) was calculated as weight in kilograms divided by the square of height in meters.

#### 2.2.2 Primary outcome: VO_2max_

The multistage 20-m shuttle run test was used to estimate VO_2max_ (Leger et al., 1988). Subjects were asked to run back and forth on a 20-m track with an audio signal. The test ended when the child failed to reach the finish line at the same time as the audio signal twice in a row or when the child stopped due to physical exhaustion. Finally, VO_2_max was then estimated using the following formula:

VO2max⁡=31.025+3.238×speed−3.248×age+0.1536×age×speed

Where *speed* is that corresponding to that stage (speed = 8 + 0.5 stage no.), age is in years.

#### 2.2.3 Secondary outcomes

To test for secondary outcomes, we used the standing long jump, vertical jump, handgrip strength test, 30-s sit-up test, and 50-m sprint. The standing long jump has been shown to be a valid assessment of explosive power (Ruiz et al., 2011). Participants were asked to swing their arms and jump forward onto the floor, where the distance was marked. Children performed three jumps, and the optimal length of the jump, measured in centimeters, was selected as the result.

The vertical jump is a valid measure of muscle strength and is positively correlated with bone structural strength and mineral content (Janz et al., 2021). Subjects were asked to start a downward motion and then jump as high as possible with both legs while touching a signpost with one hand. They performed one practice jump and two test jumps, 30 s apart.

To assess the health and function of adolescents’ musculoskeletal systems, handgrip strength is a trustworthy indicator (Ruiz et al., 2011; Janz et al., 2021). A Baseline pneumatic squeeze bulb dynamometer was used to gauge handgrip strength (Baseline, United States). Subjects were placed in a seated position with their elbows resting on a table. Two consecutive bilateral grip strength measurements were recorded, with the first measurement on the dominant hand. There was a 30-s rest period between each measurement to prevent fatigue. The best value of two trials was chosen for each hand.

The sit-up 30-s test assesses the muscular endurance of the trunk (Popovic et al., 2020). The subject lay on his/her back with knees bent, hands clasped behind the neck then rose to a sitting position, and finally returned to the starting position for one complete sit-up. The outcome was the number of sit-ups correctly completed within 30 s.

The 50-m sprint is used to assess the speed and anaerobic capacity (Fjortoft et al., 2011). On the command “Start”, the child standing behind the starting line had to run as fast as possible to the end of a 50-m track. The experimenter timed the tests with a stopwatch used in the track race (Casio, Japan), which was accurate to 0.01 s.

#### 2.2.4 Evaluation measures

An extensive process evaluation was conducted to assess the feasibility and fidelity of the intervention. The intervention center faculty completed daily monitoring reports of the activities to ensure the quality of the interventions. Also, subjects were randomly selected from the intervention and control groups to wear an ActiGraph wGT3X-BT accelerometer (ActiGraph LLC, Pensacola, FL, United States) for 1 week during the intervention, for the purpose of assessing differences in physical activity levels, and patterns between groups.

The accelerometer was attached to a wrist strap and subjects were required to wear the accelerometer on the wrist of the non-dominant hand for 7 consecutive days, including 2 weekend days for at least 10 h a day except when swimming or bathing (Clevenger et al., 2019). The subjects had to wear the accelerometer before the test to ensure that it was being worn correctly. If the participant’s accelerometer collected valid data for more than 3 days (including 1 weekend day and 2 working days), it was included in the final data analysis. A total of 90 subjects, 30 in each group, wore accelerometers. The data for 20 subjects (4 in the B-HIIT group, 9 in the R-HIIT group, and 7 in the control group) were excluded due to incomplete accelerometer data records, resulting in valid data for 70 participants. ActiLife v6.13.3 software was used to initialize, download, and calculate physical activity data. Non-wear time, defined as ≥ 90 consecutive minutes of 0 counts, was removed by the software (Choi et al., 2011). Cumulative activity counts were categorized by intensity into sedentary time (<100 counts/minute), light physical activity (101–2,799 counts/minute), moderate physical activity (2,800–4,000 counts/minute), and vigorous physical activity (>4,000 counts/minute) based on the cutoff point set in ActLife.

### 2.3 Description of intervention

Throughout the 12-week study period, the intervention group participated in two 45-min regular PE classes per week, conducted by the school’s PE teacher and following the regular curriculum. The R-HIIT and B-HIIT groups received the intervention from two trained and qualified physical education teachers, and two topic experts oversaw the procedure to ensure that the intervention was carried out correctly. The intervention group replaced the warm-up exercises in the regular program with the proposed HIIT training session and then continued to complete the planned normal PE session.

HIIT sessions were performed during the first 10–15 min of each class, including a short warm-up, with a total of 8–10 interventions, using a 2:1 (1 min:30 s) work-to-rest ratio, with exercise intensity controlled at 75%–85% of maximum heart rate. A Mio Alpha heart rate monitor (Mio Alpha, United States), was used to monitor heart rate changes per second during exercise. We utilize the computer to track changes in the subjects’ heart rates as reported in real time by the heart rate monitor. When a subject’s heart rate falls short of the target heart rate, the research team members would remind them to intensify their exercise intensity in order to reach the desired heart rate. A work-rest ratio of 2:1 is optimal for male and female participants in HIIT (Laurent et al., 2014), and exercise intensity of 75%–85% HRmax has been shown to generate positive emotions and contribute to adherence to a physical activity regimen (Malik et al., 2019). We chose the heart rate monitor because we considered it a more objective measure of exercise intensity than those used in most previous studies, which used a work-rest ratio of 1:1 and SPRINT as the mode and chose either all-out training or a percentage of maximal aerobic speed as a measure of exercise intensity (Paulino da Silva Bento et al., 2021; Solera-Martinez et al., 2021; Cao et al., 2022b; Bossmann et al., 2022; Lubans et al., 2022).

**FIGURE 1:**
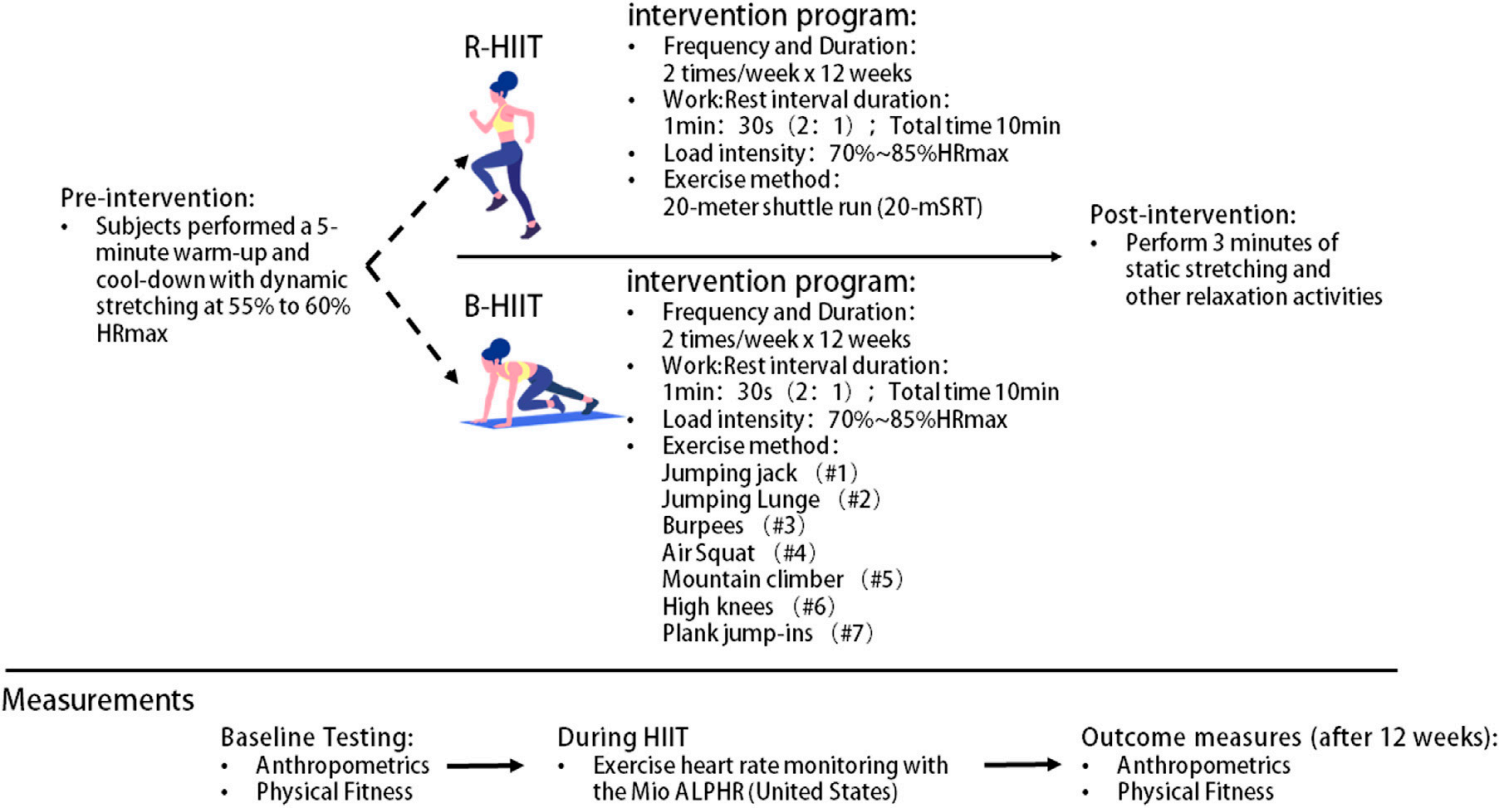
Summary of study design. Note: R-HIIT: Running-based high-intensity interval training; B-HIIT: Body weight-based high-intensity interval training.

The specific intervention for the R-HIIT group was a “real-world” training program based on a 20-m shuttle run test called “beep training” (Leger et al., 1988; Gripp et al., 2022). A flat surface was first chosen to allow the placement of two cones with a distance of 20 m between them. Each participant’s maximal aerobic speed was used to design sound cues, placing participants with the same maximal aerobic speed in groups so that they could listen to pre-recorded sounds to maintain the correct running speed. Each time they heard a “beep” sound in the music, they turned and completed a full cycle of running from the starting point to the turnaround point and back. After 4 weeks, the frequency of the musical rhythm will be accelerated according to the participants’ improved motor ability Figure 1.

The PE teacher led the B-HIIT group in performing the full-body resistance exercises along with the rhythm of the music. Movement patterns included jumping jacks, jumping lunges, burpees, air squats, mountain climbers, and high knees. After 4 weeks of the experiment, as the subjects’ training heart rate improved relative to the target heart rate, movement frequency was increased according to the improvement in the students’ exercise ability Figure 1.

Throughout the 12-week intervention period, the control group participated in regular 45-min PE classes twice a week, following a normal curriculum with the PE teacher.

The present study had high fidelity. Monitoring reports and researcher observations of the intervention groups showed that, with very few exceptions, the participants adhered to the weekly unit plan and a daily instructional plan developed in the study and controlled the intensity of the intervention through heart rate monitoring. The total exercise time and HIIT intervention time remained consistent for each group. Attendance averaged over 90% for each session.

### 2.4 Statistical analysis

We used other relevant studies to estimate the number of subjects needed for the experiment. Based on previous research (Yin et al., 2020), we set the test efficacy (1-β) to 0.80, the incidence of type I error α to 0.05, the correlation between pre-and post-intervention to 0.80, and the effect size to 2.68 (VO_2max_). The sample size required for each group was calculated to be 20. Considering a 20% dropout rate, we established the minimum number of subjects in each group as 22.

This study adopts the principle of intention-to-treat analysis. The collected data were analyzed using IBM SPSS Statistics 26 software. All data were represented as mean ± SD, and the normal distribution test and variance homogeneity test were carried out by the Shapiro-Wilk test and Levene test, respectively. One-way analysis of variance (age, height, weight, BMI) and Chi-square test (gender) was used to test the differences in demographic variables. Sex, age, and BMI are controlled as covariates. The 3 (group: R-HIIT, B-HIIT, and control) × 2 (time: pre- and post-test) repeated measurement analysis of variance was used to test the difference before and after intervention for the experimental groups. When the interaction of group × time or the main effect of time and group was significant, a simple effect analysis was used to make a statistically significant pairwise comparison (Bonferroni). In addition, the effect size with statistical significance was calculated, which was expressed as η_p_^2^, in which a small effect was 0.20, a medium effect was 0.50, and a large effect was 0.80. The significance level was set to *p* < 0.05.

## 3 Results

### 3.1 Study sample characteristics

**FIGURE 2:**
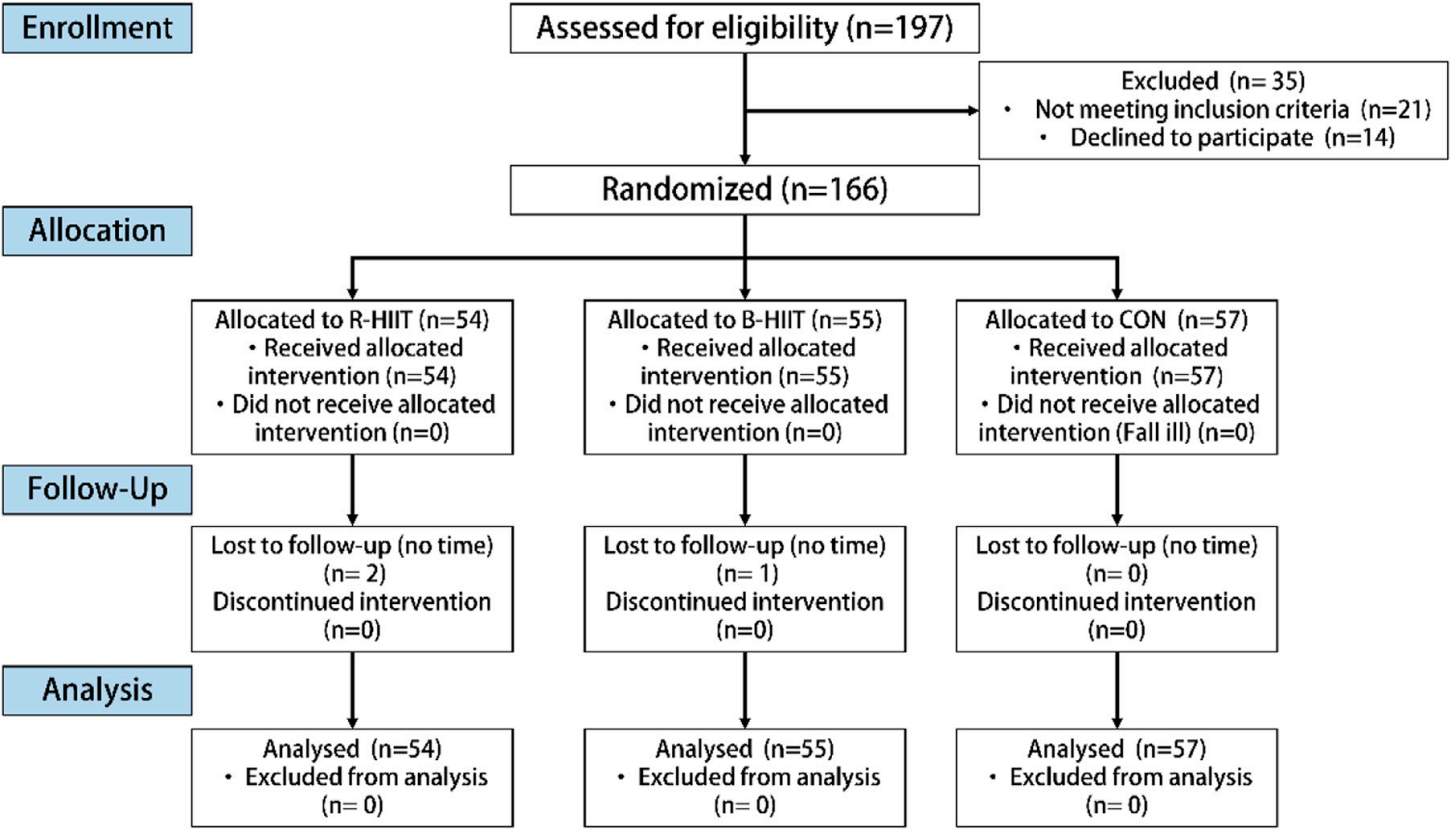
Flowchart of the participant selection process.

As shown in Figure 2, 166 of the 197 recruited subjects, or 84.26%, were selected to join the study. The other 35 subjects were excluded because they did not sign the informed consent form (*n* = 21) or were unwilling to participate (*n* = 14). Of the test subjects, 71 were male and 92 were female. No injuries were reported during the 12-week intervention period, but one male and one female dropped out of the R-HIIT group and one male dropped out of the B-HIIT group, for a total of three dropouts (1.81% rate). No significant differences were found in the baseline demographic characteristics of the two groups, as shown in Table 1. The levels of the other socioeconomic variables in the sample are shown in the Annex.

**TABLE 1:** Baseline demographic characteristics of participants (*n* = 166).

Indicators	All (*n* = 166)	R-HIIT (*n* = 54)	B-HIIT (*n* = 55)	CON (*n* = 57)	*p*
Age (year)	12.49 ± 0.31	12.47 ± 0.04	12.46 ± 0.04	12.55 ± 0.04	0.32
Sex (male/female)^a^	71/92	23/31	24/31	26/31	0.89
Height (cm)	159.20 ± 6.98	159.32 ± 7.32	159.25 ± 6.85	159.03 ± 6.91	0.97
Weight (kg)	49.98 ± 10.48	49.88 ± 9.77	50.36 ± 10.01	49.70 ± 11.67	0.94
BMI (kg/m^2^)	19.58 ± 3.13	19.53 ± 2.75	19.72 ± 2.90	19.50 ± 3.69	0.92

Note: R-HIIT: Running-based high-intensity interval training; B-HIIT: Body weight-based high-intensity interval training; CON: control group; BMI: body mass index.

^a^ Difference between the two groups tested using the chi-squared test.

### 3.2 Intervention effects

Regarding the primary outcome, repeated measures variance results showed that there was a significant interaction for VO_2max_ (F_(2,163)_ *=* 42.91, *p* < 0.001, η_*p*_^2^ *=* 0.35). A simple effects analysis showed significantly greater improvements in the mean performance of VO_2max_ in both the B-HIIT and R-HIIT groups after intervention (*p <* 0.01), as compared to the control, with the highest scores in the B-HIIT group. Regarding the mean within-group changes, both the B-HIIT and R-HIIT significantly increased the mean VO_2max_ from pre-to post-tests (*p* < 0.01), and the control group did not (*p* > 0.05) (Table 2).

**TABLE 2:** Effects of the intervention on outcomes according to group (*n* = 166).

Variables	Pretest	Post-test	The mean difference (95% CI)	η_p_^2^
VO_2max_ (mL/kg/min)				
B-HIIT	38.62 ± 3.36	43.14 ± 3.80#&	4.48 [3.89 to 5.07] **	0.69
R-HIIT	37.17 ± 3.43	41.32 ± 3.20#	3.34 [2.74 to 3.94] **	0.54
CON	38.56 ± 2.78	41.90 ± 3.59	0.75 [−0.173 to 1.33]	0.04
Standing broad jump (cm)				
B-HIIT	168.60 ± 18.53	176.45 ± 15.55##	7.97 [5.38 to 10.55] **	0.19
R-HIIT	167.45 ± 2 5.84	175.08 ± 22.47##	7.86 [5.26 to 10.51] **	0.18
CON	167.08 ± 20.94	169.93 ± 24.48	2.25 [−0.27 to 4.77]	0.02
Vertical jump (cm)				
B-HIIT	239.34 ± 12.89	247.41 ± 11.34##&&	8.10 [6.76 to 9.44] *	0.58
R-HIIT	238.28 ± 13.87	240.97 ± 14.15	2.12 [0.76 to 3.48] *	0.10
CON	242.52 ± 8.38	242.52 ± 11.13	0.05 [−1.32 to 1.33]	0.00
Handgrip strength (kg)				
B-HIIT	43.91 ± 11.57	46.76 ± 11.97	4.28 [1.76 to 2.80] **	0.16
R-HIIT	42.75 ± 9.10	45.75 ± 9.59	3.14 [1.88 to 4.39] *	0.14
CON	40.14 ± 9.25	42.29 ± 10.85	2.30 [−1.76 to 4.23]	0.10
Sit-up (CPM)				
B-HIIT	40.53 ± 7.67	45.43 ± 8.46#&	4.79 [3.35 to 6.39] *	0.30
R-HIIT	42.58 ± 9.13	42.15 ± 1.44	0.13 [−1.34 to 1.60]	0.00
CON	40.77 ± 9.62	41.54 ± 9.93	0.46 [−0.08 ± 1.73]	0.00
50-m sprint (s)				
B-HIIT	9.12 ± 0.91	8.87 ± 0.70	−0.26 [−0.39 to −0.13] *	0.13
R-HIIT	9.30 ± 1.26	8.96 ± 0.75	−0.13 [−0.27 to −0.003] *	0.04
CON	9.15 ± 0.78	9.17 ± 1.15	−0.02 [−0.11 to 0.17]	0.00

Note: VO_2max_: Maximal oxygen uptake; R-HIIT: Running-based high-intensity interval training, B-HIIT: Body weight-based high-intensity interval training. All comparisons are adjusted for sex, age, and BMI. * indicates differences in intragroup comparisons (*: *p* < 0.05; **: *p* < 0.01). # indicates intervention group compared with control group (#: *p* < 0.05; ##: *p* < 0.01). & indicates B-HIIT compared with R-HIIT (&: *p* < 0.05; &&: *p* < 0.01).

Regarding secondary outcomes, repeated measures variance results showed that the participant’s standing broad jump (F_(2,163)_ *=* 6.75, *p =* 0.01, η_*p*_^2^ = 0.04), vertical jump (F_(2,163)_ = 38.120, *p* < 0.001, η_*p*_^2^ = 0.33), handgrip strength (F_(2,163)_ = 11.51, *p* < 0.001, η_p_^2^ = 0.21), sit-up (F_(2,163)_ = 16.81, *p* < 0.001, η_*p*_^2^ = 0.18) and 50-m sprint (F_(2,163)_) = 11.063, *p* < 0.001, η_*p*_^2^ = 0.11) had significant interaction effects. The simple effects analysis showed significantly greater improvements after intervention in the mean performance of standing broad jump (*p* < 0.01) in both the B-HIIT and R-HIIT groups, and vertical jump (*p* < 0.01) and sit-up (*p* < 0.05) performance in the B-HIIT, as compared to the controls. In addition, the mean scores for vertical jump (*p* < 0.01) and sit-up (*p* < 0.05) were significantly greater in the B-HIIT group than in the R-HIIT after the intervention. Regarding the mean within-group changes from pre-to post-tests, with the exception of sit-ups which had significant improvement (*p* < 0.01) only in the B-HIIT group, both the B-HIIT and R-HIIT significantly increased the mean scores for standing broad jump (*p* < 0.01), vertical jump (*p* < 0.05), handgrip strength (*p* < 0.05), and 50-m sprint (*p* < 0.05), but not the control group (*p* > 0.05) (Table 2).

**FIGURE 3:**
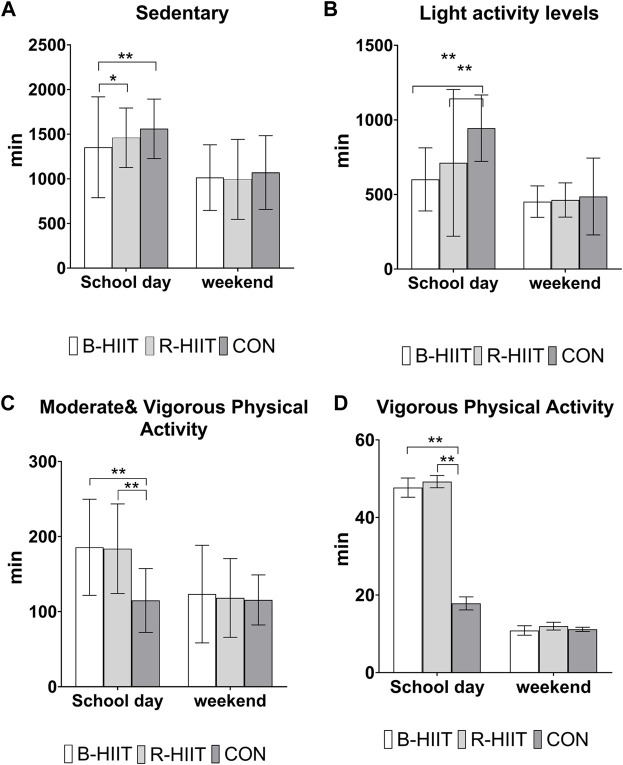
Mean weekly physical activity levels during the intervention. Note: R-HIIT: Running-based high-intensity interval training; B-HIIT: Body weight-based high-intensity interval training; CON: Control group.

Measurement of weekly physical activity levels during the intervention showed that adolescents participating in the B-HIIT and R-HIIT groups had significantly lower levels of sedentary behavior and low activity (Figures 3A, B) on school days compared to the control group (*p* < 0.05). As shown in (Figures 3C, D), the intervention groups also had significantly longer periods of moderate and vigorous or vigorous physical activity levels than the control group (*p* < 0.05). There was no significant difference in physical activity levels between the three groups at the weekend (*p* > 0.05).

## 4 Discussion

In this study, both HIIT protocols based on the school environment were successful in improving indicators of adolescent cardiorespiratory fitness and muscular strength, and the intervention group engaged in more moderate to vigorous exercise than the control group did throughout the intervention period. However, it was also discovered that the B-HIIT group, which relies on body weight alone, is more efficient than the R-HIIT group, which relies on running, at enhancing CRF, explosive leg strength (vertical leap), and abdominal muscular endurance (Sit-up). Hypothesis 1 of our study has been confirmed, but hypothesis 2 of our study is not true.

Research has shown the ability of R-HIIT in the school setting to improve aerobic endurance (Bauer et al., 2022; Harnish et al., 2022). Our study found that B-HIIT also improved cardiorespiratory fitness in adolescents, which is consistent with other studies (Paulino da Silva Bento et al., 2021; Bossmann et al., 2022). Cardiac adaptation through HIIT training can explain why resistance exercises based on body weight can lead to similar aerobic fitness as running-based programs. As long as the intensity is high enough, the type of training can be adjusted according to the sports environment of the school. However, there are contradictory findings. Engel et al. (2019) did not find that classroom-based B-HIIT sessions improved variables associated with aerobic endurance. This may be because the intervention time (6.0 ± 1.5 min) and duration (4 weeks) were short and therefore did not elicit sufficient stimulation to improve CRF. Braaksma et al. (2018) suggested a physical activity intervention for a minimum duration of 6 weeks to improve CRF in children aged 6–12.

Previous studies of children and adolescents in and out of school have found that when R-HIIT is used, the improvement in aerobic endurance is similar to or slightly lower than that of MICT, depending on the participants’ initial health level and the duration and design of the intervention (Yin et al., 2020; Cao et al., 2022a; Bauer et al., 2022). However, the present study further found that B-HIIT improved CRF better than R-HIIT. The difference value reached 2.32 mL/kg/min. Previous adult data show that each unit reduction in CRF increases the risk of all-cause mortality by 1.73 times, the risk of CVD by 2.27 times, and the risk of cancer by 2.07 times (Imboden et al., 2018). Surveys in adolescent populations have found that CRF is strongly associated with adolescent overweight/obesity, and cognitive control, and is predictive of future CVD health (Perez-Bey et al., 2019; Wisnieski et al., 2019; Shigeta et al., 2021; Gonzalez-Galvez et al., 2022). Therefore, the results of this study have encouraging clinical implications.

Various peripheral and central physiological factors could explain the elevated VO_2max_ after HIIT intervention (Bossmann et al., 2022). In this study, the possible reason why B-HIIT is superior to R-HIIT is that B-HIIT requires the recruitment of more fast muscle fibers as well as the mobilization of all types of muscle during exercise, thus leading to more blood lactate and consequently to an increase in VO_2max_ (Binzen et al., 2001). Some researchers hypothesize that resistance training can indirectly improve endurance by increasing neuromuscular adaptations, improving factors such as running economy and specific muscle output power (Paavolainen et al., 1999).

Traditional HIIT reflects time efficiency in improving several health markers and aerobic/anaerobic performance but ignores the impact on muscle health, a critical factor in the present and future health of children and adolescents (Janz et al., 2021). Costigan et al. found that running-based HIIT had a small overall effect on muscle health in young adults (Costigan et al., 2015). Requirements for a healthy musculoskeletal system include absolute muscle strength, endurance (fatigue tolerance), and the ability to produce power quickly or explosive muscular power (Plowman, 2014). In this study, R-HIIT was able to improve muscle strength in standing, broad jump, vertical jump, and handgrip strength to some extent, but not nearly as much as B-HIIT. Only B-HIIT improved core muscle endurance, as indicated by the sit-up results. This is probably because exercises like mountain climbers in the B-HIIT program improve endurance in the core lumbar and abdominal muscles. Related studies propose that the improvement of core muscle endurance can stabilize the lumbar spine and reduce the risk of lower back pain (Janz et al., 2021).

One study concluded that the increase in muscle strength after interval training could be explained by increased activity of anaerobic glycolytic enzymes in the skeletal muscle (Glaister, 2005). Most B-HIIT exercises are plyometric exercises, which are more effective than running in developing muscle mass and increasing anaerobic glycolytic enzyme activity in skeletal muscle. This, in turn, significantly improves muscle strength and explosive power (Costigan et al., 2015; Usman and Shenoy, 2019), thus explaining why B-HIIT is more effective than R-HIIT. Other research found that increased lower body strength and VO_2max_ facilitate increased stride frequency and length during running (Benedetti et al., 2018). These results may explain improved speed scores in activities such as the 50-m sprint, and they further support the use of resistance training B-HIIT for improved health and endurance. One study found that even a short 4-week B-HIIT intervention was effective in improving health-related indicators in children and adolescents (Engel et al., 2019).

Some studies have suggested that physical activity levels during one part of the day may lead to a compensatory decrease in physical activity levels during another part of the day, a hypothesis known as the “activityStat hypothesis” (Gomersall et al., 2013; Beck et al., 2022). Data from the accelerometers in our study found encouraging results to the contrary: both HIIT groups had significantly higher levels of moderate to vigorous or vigorous physical activity than the control group during the intervention period. Popowczak et al. (2022) also found that adolescents were more active on days when they performed HIIT than on other days. Ricci et al. (2020) concluded that while maximal heart rate and significant lactate accumulation were induced during HIIT, the affective value and enjoyment of exercise remained positive for most participants. Because enjoyment of exercise is a strong predictor of habitual exercise in children, this suggests that embedding HIIT into school PE classes could also help increase daily activity in children and adolescents.

Our study highlights the potential for embedding HIIT into school PE classes to improve the health of adolescents, and evidence supports the use of B-HIIT as more effective than R-HIIT for improving physical performance. From a practical perspective, B-HIIT training does not require any barbells, equipment, or machines compared to traditional apparatus-based strength training, and all exercises are performed with the children’s own body weight and at fairly high speeds and repetitions. The physical improvement after the intervention represents a good cost-benefit ratio. Furthermore, because B-HIIT has minimal space requirements, it could also be useful for students who are isolated at home due to the pandemic, with online coaching provided by teachers.

### 4.1 Study strengths and limitations

One major strength of the study was that the interventions were implemented in a middle school to compare the effectiveness of two HIIT protocols. The results can serve as a reference for selecting effective HIIT programs, including such factors as load time and intensity, to improve health outcomes. Another strength is that it can be scaled for translation because the HIIT program can be integrated into middle-school PE classes, and the efficiency of HIIT programs used in schools has been observed in improve the students’ physical fitness. Lastly, the present study objectively measured the internal load of HIIT program by monitoring HR.

Our study had some limitations. First, because it was implemented in school PE classes, it was not possible to completely randomize each student. However, as in previous studies in a school setting (Bossmann et al., 2022), minor differences in performance ability between the three groups are expected. Second, the two HIIT protocols were not evaluated in this study for affective (i.e., sensory state) and enjoyment responses in adolescents, and these variables should be evaluated in future studies, as enjoyment is a relevant motivating factor for habitual activity and exercise adherence throughout the lifespan (Ricci et al., 2020). Moreover, the relatively small number of study subjects and the fact that some subjects refused to join the study at the beginning may have led to selection bias in the study results. Finally, the subjects in this study were normal adolescents, and differences in the effects of the two interventions on the body composition of obese adolescents are not yet clear. Future school-based studies with larger sample sizes of students with different characteristics are needed to verify the effects of different HIIT interventions.

## 5 Conclusion

This study compared the efficiency of two HIIT training programs to improve the CRF of adolescents in middle school PE classes. The effect on CRF of the B-HIIT program, which consisted of resistance-based exercises using the participant’s own body weight, was significantly greater than that of the running (R-HIIT) program. This study suggests that just 10 min of HIIT training twice a week in the limited setting of school PE classes can effectively improve the CRF and muscle strength of adolescents.

## Data Availability

The original contributions presented in the study are included in the article/Supplementary Materials, further inquiries can be directed to the corresponding author.
